# Supplementary figures and images for: National trends in emergency department closures, mergers, and utilization, 2005-2015
Source: PLoS One. 2021 May 20;16(5):e0251729. doi: 10.1371/journal.pone.0251729 (PMC8136839; doi:10.1371/journal.pone.0251729)

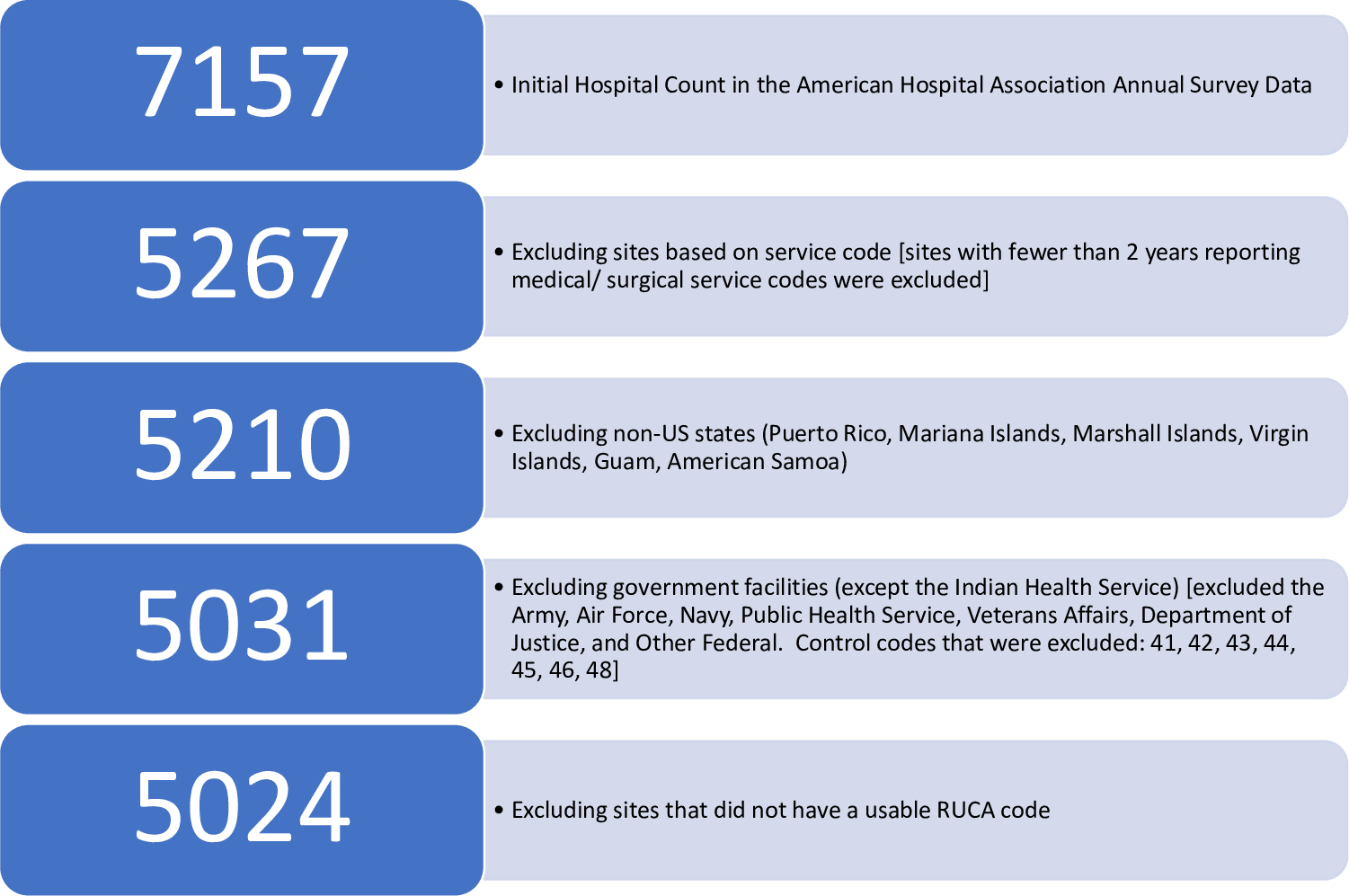

Supplement: S1 Fig — Source/Notes: Data from the American Hospital Association Annual Survey data. (TIF) [file pone.0251729.s001.tif]
